# University of Louisville

**Published:** 1847-10

**Authors:** 


### UNIVERSITY OF LOUISVILLE.

From the number of students in attendance on the preliminary course of lectures now in progress at the University of Louisville, the professors, we believe, look for an unusually large class in November. The anatomical rooms are open, and quite a number of students already engaged in practical anatomy.

